# Identifying the Qualities of Attention and the Attentional Style in Indoor Team Sports: A Gender Comparison

**DOI:** 10.3390/brainsci14070623

**Published:** 2024-06-21

**Authors:** Adela Badau

**Affiliations:** 1 Department of Motor Performances, Faculty of Physical Educational and Mountain Sports, Transilvania University of Brasov, 500036 Brasov, Romania; adela.badau@unitbv.ro; 2 Faculty of Sciences and Letters, “George Emil Palade” University of Medicine, Pharmacy, Sciences and Technology, 540142 Targu Mures, Romania

**Keywords:** attention style, external and internal dimensions of attention, gender, sport performance, qualities of attention, team sports

## Abstract

Attention is an essential psychological component in sports games, which conditions sports success. The purpose of this study was to identify the attention style (internal or external) and the weight of attention qualities depending on the practiced team sport (basketball, volleyball, or handball) and gender (female or male), in athletes aged 15–18. A total of 177 active athletes (87 female (mean age ± standard deviation: 16.07 ± 0.94 years) and 90 male (mean age ± standard deviation: 15.96 ± 0.82 years)) were involved in the study, including 62 handball players (28 female and 34 male), 58 volleyball players (30 female and 28 male) and 57 basketball players (29 female and 28 male). In the study, two questionnaires were applied: one implemented to identify the attentive style with the two dimensions internal and external (standardized), called questionnaire for the assessment of attentional style in athletes (QASA), and one designed by us, called questionnaire to identify the weights of attention qualities according to the characteristics of the practiced sport (QAQCS), aiming to identify the most relevant quality of attention, depending on the practiced sport. Cronbach’s alpha for both questionnaires was between 0.701 and 0.855. The results recorded in the present study reveal a variation in the attentional style between the groups of athletes and between genders. The results of the study highlight that in handball, girls have a predominantly external style with a total of 10.213 points; in boys’ handball, the predominant focus of attention is internal with a total score of 9.087 points. Girls’ volleyball focus of attention is predominantly external, with 8.999 points; in boys’ volleyball, the attention style is internal, registering a score of 9.713 points. In girls’ basketball, the predominant focus of attention is internal, with a total score of 8.516 points; in boys’ basketball, the external attention style is predominant, with 9.213 points. Looking at the weight of attention qualities, it was found that the most relevant for girls is concentration and mobility for handball players, stability was identified in volleyball, and distributiveness in basketball. In boys’ handball teams, mobility is the most essential, just like in basketball, and in volleyball it was found that stability has the biggest impact. ANOVA analysis highlighted statistically significant differences between groups of sports games by gender category, at both subscales of (QASA), as well as QAQCS, *p* < 0.05. The results of our study highlight differences between the attentional styles and their qualities in relation to the gender and the specific sport played, which determines differentiated approaches to these psychological components.

## 1. Introduction

Psychological preparation is an important component in the optimization of sport performances and identifying the psychological characteristics and peculiarities of athletes requires special attention in order to make the methodology of sports training more efficient. Sporting success requires the optimization of all an athletes’ potentials, namely physical, functional, psychological, technical, and tactical. Attention is an essential component in practicing team sports games and identifying the style and qualities of attention facilitates the optimization of sports training [1,2]. 

Practicing sports games requires the development of some psychological skills: concentration, attention, mental imagery, emotional control, will, etc. [3,4]. The sports training process is a multidimensional and dynamic process focused on the established performance objectives [5,6]. 

In the case of team sports games, the impact of sports psychology on the awareness and development of the athletes’ psychological skills, in which attention plays an essential role, can contribute to the improvement of individual performance, with connotations on team performance [7,8,9]. Depending on the complexity, the level of performance, the strategy approached and the technical–tactical level of the athletes, the cognitive demands during games or sports training require higher indices in information processing, as well as a high level of the ability to concentrate and pay attention [10,11,12]. The optimization of the mental processes determines the maximization of the performance of the players in the sports confrontations by adapting the sports behaviors on the anticipatory tactical bases of the action of the opponents and the game strategies of the opposing team [13,14]. 

Team sports games are characterized by diversity and specificities regarding the number of players, the playing surface, the game rules, etc. Athletes’ ability to concentrate is influenced by a series of specific, internal or external stimuli [15,16,17]. The mental pressure to which athletes are subjected in training and competitions is determined both by internal stimuli, such as anticipating the movements of teammates or opponents, the desire for success and victory, positive emotions, fear of failure, etc., as well as by external stimuli: the audience, the quality of the field, the characteristics of the equipment, etc. [18,19]. According to specialists in the field of sports psychology [20], the most demanding psychological component in sports games is attentional concentration, which represents a complex process based on two types of information processing: conscious and unconscious [21,22]. Controlled or conscious informational processing requires physical and intellectual efforts from athletes, while involuntary or unconscious informational processing involves the athletes execution of the task without making a conscious effort but with a precise and clear focus [13,22,23]. Conscious processing is specific to the stage of learning, repetition of a motor skill, and unconscious processing takes place mainly in the stage of consolidation, perfection of motor skills, which over time, through repetitions, becomes automatic [19,24].

To describe attentional processes from the perspective of cognitive science, several terms are used: attention, concentration, focus, etc. Attention refers to the ability to selectively process certain information by directing cognitive resources to specific stimuli or tasks [25]. Focusing attention aims to direct mental effort on a certain aspect or characteristic of a stimulus [26]. There are two types of attention: selective attention, which is the ability to select a stimulus for focus in the presence of distractions, and divided attention, which is the ability to simultaneously focus on two or more things, performing two skills simultaneously [27]. Nideffer (1998), a sports psychologist, considers that athletes have a tendency to change their attention style in relation to the sport practiced and depending on the targeted performance objective [28]. The focus of attention has two dimensions: direction (internal–external) and width (wide–narrow) [29]. The internal and external directions of the focus of attention aim at an introspective and extrospective perspective, respectively. The second dimension, width, has an integrative (expansive) orientation, the selective extreme. Previous studies found that at the level of sports initiation, the attentional external style predominates [20,30], and at the level of high performance, complexity and variance are predominant, depending on the experience in sports and social and biological factors [31,32].

Taking into account the difference in the focus of attention on gender, a series of studies did not highlight significant differences between individual sports and team sports [33,34]. Studies have found that female athletes have a predominance for internal attention, aiming at the motor task [35,36,37], and aesthetic motivation compared to male athletes, who are motivated by the competitive attitude, having a tendency to focus external attention [36,38].

The study of attentional mechanisms, in conjunction with the psychological factors underlying motor learning, has been a concern of specialists who have devised a series of specific theories. The theory of information processing is known as the constrained action hypothesis by Wulf et al. (2021) [39], in which it was considered that focusing internal attention on the biomechanics of a movement can disrupt automatic movement planning/execution. The OPTIMA theory (performance optimization through intrinsic motivation and attention for learning) of Wulf and Lewthwaite [39,40], focuses on a holistic approach, through which learning is a consequence of the interactions between attentional and motivational factors. It is suggested that in ideal sensorimotor and motivational conditions, aimed at associating the goal with the action, functional connections are more effective in the brain networks. In sports activities, the promotion of learning based on autonomy in correlation with performance expectations and with an external focus direction can facilitate dopaminergic responses and optimal involvement in the motor task [39,40]. A recent approach, called the ecological dynamics account of attentional focus [41], takes into account the characteristics of the environment in relation to the direction of attentional resources, evidences that environmental stimuli can contribute to the improvement in the following: motor self-organization; decision-making capacities; and awareness and predicting the movements of teammates and opponents [42,43]. The previously mentioned theories explain the dynamics of the focus of attention in the preparation process aimed at obtaining sports performances; thus, the internal attention is correlated with the automatic movements that become more effective if they are left unattended by an external focus [39]. The recent literature [44,45] shows some reticence regarding the theory of the constrained action hypothesis [39] and the OPTIMAL theory [40].

Among the main qualities of attention, we identify the following: stability, volume, intensity (concentration), mobility, distribution, and distraction [46,47]. Stability of attention comprises the long-term maintenance of orientation and concentration on the same object or on the same activity. The intensity consists of a focus of internal excitation, and the nearby areas are relatively inhibited and inaccessible to disturbing factors. The volume of attention represents the number of objects or phenomena that can be included simultaneously in the field of clear reflection. Mobility or flexibility of attention is the ability to move attention from one object to another in short time intervals. The distribution of attention is characterized by the number of activities that a person can perform simultaneously without one interfering too much with the others. Distracting attention targets stimuli with a certain intensity that causes external inhibition mechanisms in the activity in which the person is involved [48,49]. In the process of theoretical psychological training, an important focus is on the ability to ignore distractions, which will significantly involve the ability of mental attention and concentration [50].

The specialized literature that address the qualities of attention are limited. A study carried out by Stavrev and Ivanov (2019) [51] aimed at the qualities of stability and concentration of attention in the game of university basketball and volleyball, did not find significant differences between the sports. Some studies have focused on the impact of one or two qualities of attention in field tennis and in recreational aerobic gymnastics programs, among students from the academic environment [52,53], in boxing [54], and in combat sports [55]. Based on the study of the specialized literature, we have not identified any study that addresses the identification of the impact of attention qualities depending on the team sport practiced and gender, at the level of active athletes aged between 15–18 years. The purpose of this study was to identify the attention style (internal or external) and the weight of attention qualities depending on the team sport practiced (basketball, volleyball, or handball) and gender (female or male), in athletes aged 15–18.

H1—The hypothesis of the study started from the assumption that the focus of attention is influenced by the practiced team sport and gender, in athletes aged between 15 and 18 years.

H2—The hypothesis started from the assumption that the impact of attention qualities is different depending on the team sport practiced and the type of athletes.

## 2. Materials and Methods

### 2.1. Participants

Before starting the statistical procedures for verifying the formulated hypotheses, it is necessary to carry out some procedures regarding the power of the test and the size of the effect. Power analysis integrates a set of procedures aimed at identifying fundamental parameters regarding the scientific relevance of particular methodological approaches such as: effect size, alpha, type II error, sample size, variability of distributions and, of course, power [56,57]. The power analysis was performed with the program G*Power 3, offered free-of-charge to users (open-source) by the Institute of Experimental Psychology at the Heinrich Heine University in Dusseldorf, version 3.1.9.7.

**Figure 1:**
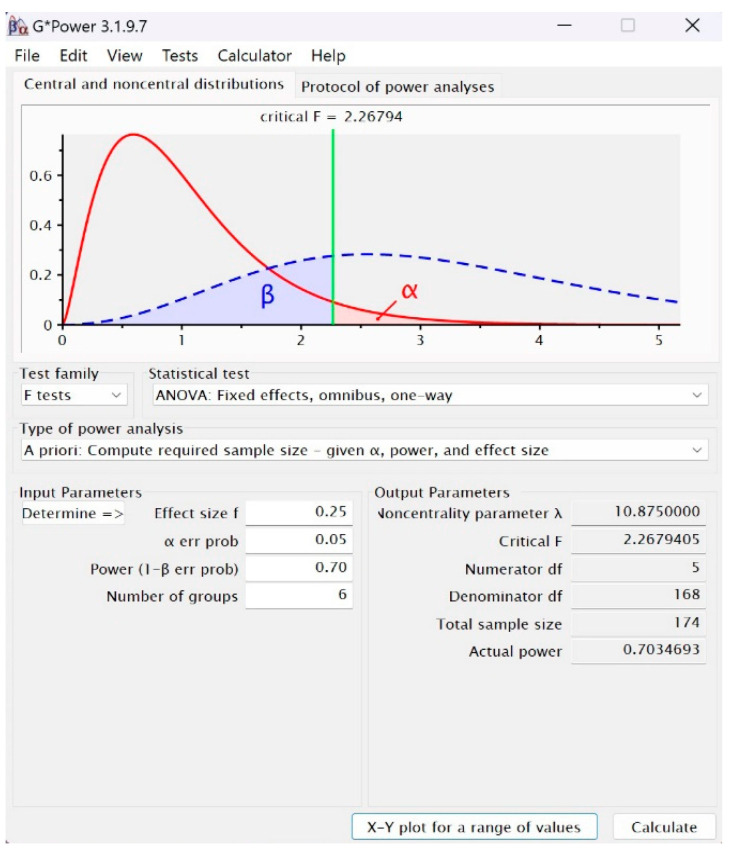
Power analysis—input and output parameters.

From the perspective of the type of power analysis, the “A priori” method was selected, a method that provides relevant information regarding the volume of the sample/samples depending on the desired level of test power, the type 1 error level (alpha), and the effect size. The values of the input and output parameters can be seen in Figure 1.

It is observed that the critical F has a value of 2.267 at an approximate power of 0.70, which corresponds to a type II error of 0.3 and is minimally accepted [58,59,60], and a minimum sample size of 174 for the 6 groups entered. The volume of the sample included in the research is 177. The graph in the upper part of Figure 1 illustrates the ratio between the H0 and H1 distributions, projects the intersection of alpha and beta type errors, and indicates a limited possibility of rejecting the null hypothesis.

**Figure 2:**
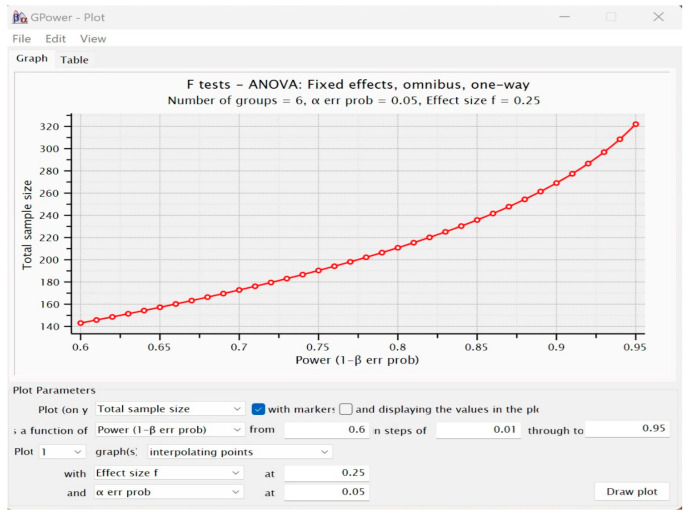
Variation of research power as a function of input parameters.

We believe that such minimum requirements can be acceptable under the conditions of this exploratory experimental approach. In future research, we aim to increase the power of the test and the sample size included in the research to significantly increase the quality of the research. The graph presented in Figure 2 shows the variation of power under the conditions of the input parameters for the different sample volumes and will represent a benchmark for increasing the quality of future research.

The present study included a total of 177 registered active athletes from the following team sports: handball, volleyball, and basketball. The data of the study participants, by groups, are presented in Table 1.

The age and duration of practicing different sports were collected from the official documents of the sports clubs, provided by the coaches. Athletes are members of the municipal clubs in Brasov and Targu Mures (Romania) that have indoor team sports sections (volleyball, handball, and basketball); all the subjects are domiciled and come from an urban environment. The participants voluntarily participated in this study and adhered to the principles outlined in the Declaration of Helsinki and approved by the Review Board of Physical Education and Sports, UMFST Targu Mures, Romania, no. 37/29.04.2023. Inclusion criteria: active athletes, clinically healthy, age between 15 and 18 years, full completion of the questionnaires, minimum 3 years of sports experience. In the preliminary stage of this study, all participants were informed of the objectives of the study. Before applying the questionnaires, verbal informed consent was obtained from the coaches, all participants signed an informed consent, and their parents or guardians also signed an informed consent.

### 2.2. Study Design and Procedures

Observational research with a cross-sectional strategy was carried out between May and July 2023, Figure 3. The two questionnaires were applied physically, in a technical session with the agreement of the coaches (day 1) and athletes (day 2). During the technical sessions, the athletes were presented with the most relevant theoretical aspects regarding the concept of attention, the typology of the attention style, the qualities of attention, and relevant aspects regarding attention in indoor sports games (day 3). After the theoretical information sessions, the two questionnaires were applied to the athletes (day 3), with preliminary information before starting the questionnaires.

### 2.3. Assessment Tools

The study included the application of two questionnaires to the athletes practicing team games (handball, volleyball and basketball):

1. The questionnaire for the assessment of attentional style in athletes (QASA) [18], a standardized questionnaire, includes 6 items and evaluates the attention skills in the direction dimension, namely internal or external. Items 1, 2, and 6 assess the internal dimension, referring to scale A, and items 3, 4, and 5, assess the external dimension, referring to scale B. The rating scale per item was from 0 to 4, where 0—never; 1—rarely; 2—sometimes; 3—frequently; and 4—always. Internal attention focused on the following aspects: the ability to perceive what is happening on the field and with teammates; the ability to focus only on a sporting action; the ability to listen to the trainer’s instructions with a quick focus, without being distracted by one’s own thoughts, ideas, etc. The external dimension of attention includes the following: focusing on a player but having the ability to look at things as a whole; the ability to imagine a game situation from other people’s information; and the ability to know the evolution of teammates at any time.
2. The questionnaire to identify the weights of attention qualities according to the characteristics of the practiced sport (QAQCS). This questionnaire was designed by us, based on the typology of attention qualities and aimed at the incidence of attention qualities in the opinion of team sports athletes. The questionnaire included 6 items related to the 6 qualities of attention, namely: volume; stability; intensity (concentration); distributivity; flexibility (mobility); and distraction. Each item consisted in the appreciation of the qualities of attention according to the characteristics of the practiced sports game. The Likert rating scale from 1 to 5 was used for each item, thus 1—very little, and 5—very much.

### 2.4. Statistical Analysis

The statistical analysis was carried out with the SPSS 22 program, calculating the following statistical parameters: arithmetic mean, minimum (Min), maximum (Max), standard deviation (SD), and Cronbach’s alpha value (α) for the validity of the questionnaires. We used the Students t-test for the differences between groups according to gender (male and female); significance threshold selected for the study *p* < 0.05; and confidence interval for the mean (CI–95%) with lower and upper benchmarks. The mean difference between the sports game groups was analyzed using an ANOVA analysis of variance: Fisher test and multiple comparisons (LSD). To calculate the size of the effect, we used the statistical parameter Cohen’s d, where = 0.2 very small, 0.2–0.5 small, 05–0.8 medium, and >0.8 large. The percentage of variance was also calculated, which measures the proportion in which a mathematical model takes into account the variation (dispersion) of a given data set. The Kurtosis indicator was also used, which highlights the probability of the distribution of a random variable with a real value. A confirmatory factorial analysis of the newly designed questionnaire was also carried out using statistical parameters: correlation, KMO, and Bartlett’s test, effect size, and total variance explained. Power analysis was performed with the G*Power 3 program that provides relevant information on sample size in correlation with test power, type 1 error level (alpha), and effect size.

## 3. Results

### 3.1. Validity and Reliability—Questionnaire to Identify the Weights of Attention Qualities According to the Characteristics of the Practiced Sport (QAQCS)

The purpose of initiating the exploratory factor analysis procedure is to detect structure in the relationships between variables, resolve the collinearity problem, and validate the scale construct [57]. Due to the fact that the instruments were created on the occasion of this research and the factorial structure was not analyzed, the demersal is exploratory and not confirmatory. Since the wording items aim to assess the same construct, we expect a unifactorial structure in the instrument. Analyzing the correlations between the qualities of attention, according to Table 2, it is observed that the strongest correlation was registered between the qualities of attention flexibility and stability of 0.523 and flexibility with distraction of 0.420.

The value of the Kaiser–Meyer–Olkin index (0.753), as well as the significance level of the test of sphericity (185.709; sig < 0.001), suggests the existence of one or more common factors, which justifies the initiation of the factor reduction procedure (Table 3).

Adequate communality values, which represent the multiple correlation coefficients for each variable, indicate an adequate factorial model (Table 4).

According to Table 5, it can be seen that although six factors were generated, only one manages to reach the selection criterion (Eigenvalue = 1). The variance explained by the factor that reaches the selection criterion is 41.727.

Table 6, component matrix, presents the list of variables and their contribution to the factor loading. So, we are discussing a one-factor solution to the statistical approach. Due to the fact that only one factor was extracted, no further component rotation was performed.

The reliability of the two questionnaires, by calculating the statistical index Cronbach’s alpha, highlights that the reliability of the two questionnaires was high and very high, for all study groups (Table 7).

After collecting the applied questionnaires and analyzing the results, we will present the most relevant statistical indicators recorded in the study. For the assessment of attentional style in athletes (QASA) questionnaire, the analysis of the data on the two internal and external subscales, depending on the arithmetic mean recorded for the specific items, reveals the following: Girls’ handball is a predominant external style with a total of 10.213 points compared to the internal style, where only 7.998 points were registered, the difference being 2.215 points. In boys’ handball, the predominant attention style is internal, with a total score of 9.087 points; the external score registered a value of 8.44 points, with a difference of 0.647. In girls’ volleyball, the attentional style is predominantly external with 8.999 points at a difference of 0.466 points compared to the external focus of attention, which registered a total score of 8.533 points; in boys’ volleyball, the attention style is internal with a score of 9.713 points compared to the external one of 9.571 points. In girls’ basketball, the predominant focus of attention is internal, with a total score of 8.516 points compared to the score recorded for the external style of 7.688 points, the difference being 0.828 points. In boys’ basketball, the external focus of attention predominates with 9.213 points, with a very small difference of 0.072 points, from the internal style, which recorded a total score of 9.141 points. In conclusion, in handball and volleyball for girls, the external focus of attention predominates, and in basketball, the internal style. For boys, in handball and volleyball, the internal attention style predominates, and in basketball, the external one. The Kurtosis index, regarding the distribution of the data, falls within the limits of normality for all targeted items for all study groups, being between −2.160 and 1.182 (Table 8).

### 3.2. Questionnaire for the Assessment of Attentional Style in Athletes (QASA)

The ANOVA analysis of variance (Table 9) allowed us to identify the differences between the arithmetic means of the two categories of girls and boys subjects according to the internal and external attentional style, in the questionnaire applied for the assessment of the attentional style. Analyzing the ANOVA results, we find statistically significant differences between groups of sports games by gender category, of both subscales (internal attention and external attention) of the questionnaire for the assessment of attentional style in athletes (QASA), *p* < 0.05.

Making multiple comparisons (Table 10), the groups of subjects were statistically significant, *p* < 0.05. The differences of the arithmetic averages on the groups of sports games fell between the lower and upper limits specific to the 95% CI confidentiality interval, for both subscales of the attention style questionnaire. In the girls’ groups, the biggest differences in arithmetic means were recorded for Ai between volleyball and basketball, with a value of 0.328 points, and for Ae at 2.524 points. In the groups of boys, the biggest difference in averages between the groups of athletes was recorded between volleyball and basketball, with Ai at 0.642 points and Ae at 0.357 points.

### 3.3. Questionnaire to Identify the Weights of Attention Qualities According to the Characteristics of the Practiced Sport

In the analysis of the results of the questionnaire for identifing the weights of attention qualities according to the characteristics of the practiced sport, taking into account the arithmetic mean, we find that in the groups of girls handball the most representative qualities of attention are concentration and mobility, registering a value of 4.071, and the least representative is distraction with 2.353. In volleyball, the girls consider that stability is the most relevant and the least relevant was the distraction, due to the regulation that does not allow making any form of noise during certain phases of the game. In basketball, the most relevant is distributiveness, and the least appreciated is distraction. In boys handball teams, it was found that mobility is the most relevant; in volleyball, it is stability; and in basketball, it is mobility. The least significant in all groups of sports games was distraction. The Kurtosis index, regarding the data distribution, falls within the limits of normality for all targeted items for all study groups, being between −1.956 and 0.420 (Table 11).

The application of the ANOVA analysis of variance (Table 12), allowed us to identify the differences for *p* < 0.05, between the arithmetic means of the two categories of subjects, girls and boys, regarding the qualities of attention. The ANOVA analysis highlights that the differences between the female groups from the three sports were statistically significant (F = 12.430, *p* < 0.01); also for the male groups, the differences between the handball, volleyball, and basketball groups were statistically significant (F = 78.991, *p* = 0.02).

By performing multiple comparisons (Table 13), the groups of subjects were statistically significant. The differences of the arithmetic averages on the groups of sports games, fell within the limits of the confidentiality interval 95% CI, for all the qualities of attention included in the questionnaire. In the girls’ groups, the biggest differences in arithmetic means were recorded between handball and volleyball at 4.361 points, and in boys between handball and basketball at 3.129 points. The effect size calculated between the two groups according to gender recorded a value of d = 0.67, which reflects a medium size of the effect.

## 4. Discussion

The purpose of this study was to identify the attention style (internal or external) and the weight of attention qualities depending on the practiced team sport (basketball, volleyball, and handball) and gender (female and male) in athletes aged 15–18. The results of the study reveal the fact that in female groups, attention has an internal dimension in handball and volleyball and an external one in basketball, while in boys, handball players present an internal dimension and in volleyball and basketball an external one. Identifying the type of attention depending on the team sports game practiced and depending on gender, we consider that it contributes to the understanding of the role that attention plays in the optimization of sports performance. The identification of the internal and external attentional types facilitates the understanding by coaches, psychologists, and athletes of the psychic mechanisms that can affect psychological and sports training. Our study substantiates other previous studies that highlighted the role and importance of attention in sports games.

In a previous study carried out by Biscaia et al. (2021) [61], on samples of girls playing handball, Biscaia et al. applied the Nideffer attentional and interpersonal style test (AIST) questionnaire (1976), targeting the attentional style in the age category of 15–16 years, and found that they presented an external dimension registering a score of 5.00 ± 1.09 compared to the internal dimension of 4.81 ± 0.91. In the 17–18-year-old age category, the external and internal dimensions were equal with a score of 4.38. These results are in line with the results of the present study [61], where an external dimension of attention to handball girls was also recorded. A previous study regarding the attention style of senior handball players, depending on the position in the game, found that it varies, but the external dimension of attention predominates [62], a finding that differs from the results registered by us; we consider that this fact is due to the difference in experience, our subjects being juniors (with a more limited sporting experience) and not seniors.

In accordance with our study, Summers et al. (1991) found that in basketball there is a variation in the dimensions of attention from internal to external according to age and experience in both girls and boys [63]. In the case of basketball, a difference in attentional style was recorded between genders, with boys registering an external dimension and girls registering an internal. These results are different from previous findings [64,65,66]. In volleyball, a previous study by Fontani et al. (2006) [67], on a sample of boys aged 17–18 years, found a dimension of the external attention style, a result that aligns with our result; the difference was in the experience of the game, which, in the mentioned study, was under 3 years. Previous studies found that in volleyball, the predominance of attention is external, findings that align with the results of our study [21,67,68].

A review on the focus of attention in team sports found that the external dimension is predominant, having a beneficial role in obtaining performances [66], compared to the internal dimension, which affects performance [69]. Previous studies regarding the determination of attentional style were carried out in both individual and team sports, in different age categories, such as basketball, athletics, handball, shooting, golf, badminton, etc. [13,70,71,72], having the practical–methodical objective of understanding and improving the effectiveness of coaching [73]. Other studies have focused on the correlations between visual attention and reaction speed in association with the type of sport practiced; the results being statistically significant in team sports [74,75]. Attention to athletes during sports performances is dynamic; they show changes due to the variation of focus points [41,42,76,77]. Taking into account the differences between genders, aiming at the dimensions of the focus of attention, in the learning process of sports skills, Wulf et al. (2003) [78] consider that female athletes show a greater motor learning advantage when they are provided with externally focused instructions, compared to boys. The results of our study highlight that in handball and volleyball, the attention style is external; the only exception is with girls’ volleyball, and for boys, the domestic style prevails in volleyball and handball. A series of studies have focused on highlighting different aspects of attention; thus, a study conducted on basketball players found that there are no significant differences between genders regarding visual attention [79]. Reigal et al. (2022) found that the attention span of athletes who practice open sports (which varies depending on a number of factors such as the movement of opponents and teammates, etc.) is better compared to those who practice closed sports (in which the external environment does not influence performance). This finding was also replicated in the evaluation by gender [80].

Based on the analysis of the present study, we found that the qualities of attention vary depending on the sport practiced and the gender of the athletes. Thus, for the female groups, the most relevant qualities of attention were very different, which are as follows: in handball, concentration and mobility recorded an identical score; in volleyball, stability was the most relevant; for basketball, distributiveness was considered the most important quality of attention. In the male groups, handball and basketball players appreciated the mobility of attention the most, and volleyball players considered the stability of attention to be the most important.

Regarding the relevance of attention qualities depending on the practiced sport and the identification of studies that align with our results, there are very few. Stavrev and Ivanov (2019) analyzed two of the qualities of attention, namely stability and concentration, starting from the assumption that they are most representative in boys’ basketball and volleyball games, they found that concentration is significant in both groups, in both sports, while the stability in the basketball game did not present a good homogeneity [54]. Another study conducted on 30 girls who play handball (mean age = 14.33 years, SD = 0.48), analyzed the mobility and concentration of attention in correlation with sports performance by applying a linguistic intervention program, and found that there are significant positive correlations between the investigated intellect dimensions (analogous transfer and attention mobility) and preferential status index values [55,56]. Flexibility of attention, according to our study, is the quality with the greatest impact on handball in both genders, as well as in boys basketball. Flexibility (mobility) is conditioned by an optimal level of activation in order to dispose of all attentional resources, and motor skills have a high degree of automation [18,81], aspects that correlate with the experience of our subjects. Hutterman et al., in a study on motor response, found that players in team sports are more concerned with improving their attentional concentration and making correct decisions compared to those who practice individual sports, a finding that aligns with the results of our study regarding the flexibility and stability of attention [82]. Analyzing the specialized literature, we found that most of the studies carried out on the qualities of attention are focused on medical fields, targeting mental deficiencies, and were not focused on healthy people or on athletes. In this frame of thought, we consider that the present study presents an essential contribution to the understanding in the way in which the qualities of attention are appreciated by athletes from different team games. Based on the results of our study, we believe that specialists can better understand the mechanisms of attention depending on the characteristics of the practiced sport and will be able to optimally guide the psychological preparation process with new scientific information. Distraction of attention in all groups of subjects recorded the lowest values. We consider that this aspect is due to the game experience that offers the athletes the ability to pay attention to the phases of the game and the dynamics of the game, processed both in the preparation process and in the official matches.

Limitations in the study include the following: a relatively small number of subjects by sports and gender categories; a lack of an interventional program; the study included only subjects between the ages of 15 and 18, and athletes in other categories of greater or smaller age were not taken into account; athletes who practice outdoor team sports (e.g., football and rugby) were not included in the study; the influence and dynamics of environmental factors that could have modified the identification of focus of attention and qualities of attention were not targeted. Since the study is cross-sectional, the dynamics of the focus of attention over a certain period of time were not identified, which would have facilitated the identification of the variation in the quality of attention and the changes in the focus of attention. The application of questionnaires with evaluation scales only allows the identification of individual answers regarding the defined concepts without allowing descriptive arguments, which could highlight changes in concept and construct. Another limitation of the study is the fact that the socio-economic, educational, and geographical level of the subjects was not taken into account because, in Romania, the training of athletes registered at municipal clubs is free; school education is compulsory and free until the completion of high school studies (18–19 years).

Based on the findings of this study, future research could be directed towards identifying the dynamics of attentional focus depending on the competition stages. Another possibility would be to identify if the relevance of attention qualities changes depending on the performance objectives, as well as identify the qualities of attention relevant to the preparation process and apply some interventional strategies in order to better understand the characteristics of the sports context in order to obtain more effective individual results. Moreover, qualitative investigations provide us with details regarding the role of intra-personal variables, such as attentional focus and qualities of attention, in the context of team sports and environmental and social factors. It is an economical and reliable research method.

## 5. Conclusions

The results recorded in the present study reveal a variation in the focus of attention between groups of athletes and between genders. In the age category of 15–17 years for girls, an external dimension of attention was recorded in handball and volleyball and an internal one in the game of basketball. For boys, the dimension of attention was internal in handball and volleyball and external in basketball. Looking at the weight of the qualities of attention, it was found that the most relevant for girls are concentration and mobility for handball players; stability was identified in volleyball; and distributiveness in basketball. In boys handball teams, the most essential is mobility, just like in basketball, and in volleyball, it was found that stability has the biggest impact. The interdisciplinary approach to sports performance is a condition and a current trend of scientific research. The theoretical and practical implications found in the study will allow the optimization of the sports training methodology from the perspective of psychological training, with direct implications for physical, technical, and tactical training, in order to obtain relevant performance. Sports performance requires specialists and athletes to focus their training on all components of sports training, and the knowledge of psychological skills facilitates sports success.

## Figures and Tables

**Figure 3:**
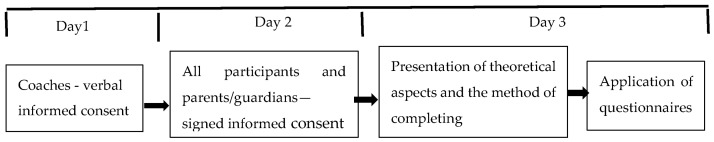
Research design.

**Table 1:** Data on the distribution of subjects, age, and experience.

Groups	Number of Subjects	Age—Years			The Experience—Years		
		(X ± SD)	Min.	Max.	(X ± SD)	Min.	Max.
Girls handball (HG)	28	16.64 ± 1.51	15	18	5.78 ± 2.05	3	9
Boys handball (HB)	34	15.94 ± 0.66	15	18	5.41 ± 1.83	3	9
Girls volleyball (VG)	30	16.67 ± 0.98	15	18	6.20 ± 1.26	4	8
Boys volleyball (VB)	28	16.43 ± 0.94	15	18	4.92 ± 0.99	5	8
Girls basketball (BG)	29	15.00 ± 0.77	15	18	5.08 ± 1.46	4	7
Boys basketball (BB)	28	15.57 ± 0.75	15	18	6.57 ± 0.64	5	8

X—mean; SD—standard deviation; Min.—minimum; Max.—maximum.

**Table 2:** Correlation matrix—questionnaire to identify the weights of attention qualities according to the characteristics of the practiced sport (QAQCS).

		Volume	Stability	Concentration	Distributivity	Flexibility	Distraction
Correlation	volume	-	0.319	0.234	0.237	0.468	0.175
	stability	0.319	-	0.290	0.258	0.523	0.225
	concentration	0.234	0.290	-	0.196	0.291	0.167
	distributivity	0.237	0.258	0.196	-	0.306	0.270
	mobility	0.468	0.523	0.291	0.306	-	0.420
	distraction	0.175	0.225	0.167	0.270	0.420	-
*p*	volume	-	0.000	0.001	0.001	0.000	0.010
	stability	0.000	-	0.000	0.000	0.000	0.001
	concentration	0.001	0.000	-	0.004	0.000	0.013
	distributivity	0.001	0.000	0.004	-	0.000	0.000
	mobility	0.000	0.000	0.000	0.000	-	0.000
	distraction	0.010	0.001	0.013	0.000	0.000	-

*p*—Sig. (One-tailed).

**Table 3:** KMO and Bartlett’s test.

Kaiser–Meyer–Olkin Measure of Sampling Adequacy		0.753
Bartlett’s Test of Sphericity	Approx. Chi-Square	185.709
	df	15
	*p*	0.000

df—degree of freedom and *p*—level of statistical probability.

**Table 4:** Communalities (extraction method: principal component analysis).

Component	Initial	Extraction
volume	1.000	0.413
stability	1.000	0.497
concentration	1.000	0.282
distributivity	1.000	0.314
mobility	1.000	0.674
distraction	1.000	0.324

**Table 5:** Total variance explained (extraction method: principal component analysis).

Component	Initial Eigenvalues			Extraction Sums of Squared Loadings		
	Total	% of Variance	Cumulative %	Total	% of Variance	Cumulative %
volume	2.504	41.727	41.727	2.504	41.727	41.727
stability	0.892	14.862	56.589			
concentration	0.798	13.295	69.884			
distributivity	0.753	12.549	82.433			
mobility	0.666	11.105	93.538			
distraction	0.388	6.462	100.000			

**Table 6:** Component matrix ^a^.

Items	Component 1
volume	0.642
stability	0.705
concentration	0.531
distributivity	0.561
mobility	0.821
distraction	0.569

^a^ Components extracted.

**Table 7:** The reliability of questionnaires on groups of subjects—Cronbach’s alpha.

Sports	Groups	Questionnaire for the Assessment of Attentional Style in Athletes (α Cronbach’s)	Questionnaire to Identify the Weights of Attention Qualities According to the Characteristics of the Practiced Sport (α Cronbach’s)
Whole questionnaire	Total sample (177 subjects)	0.787	0.709
Handball	Girls	0.742	0.730
	Boys	0.721	0.701
Volleyball	Girls	0.730	0.717
	Boys	0.840	0.805
Basketball	Girls	0.855	0.798
	Boys	0.733	0.739

**Table 8:** Descriptive statistics of the results of the questionnaire for the assessment of attentional style in athletes (QASA).

Group	Scale	Itemi	N	Min.	Max.	Mean	SD	Kurtosis
HG	Ai	I1. I am able to “read” what is happening on the field and “feel” the mood of my teammates	28	2.00	3.00	2.928	0.262	1.183
		I2. I am able to stop my thoughts while focusing on a certain event, game situation or discussion	28	2.00	4.00	3.285	0.712	−0.830
		I6. When I listen to the coach’s instructions, I am not quickly distracted from my own thoughts or ideas	28	0.00	3.00	1.785	0.956	−1.070
	Ae	I3. Even if I focus on what a certain player is doing, I manage to look at things as a whole	28	3.00	4.00	3.500	0.509	−2.160
		I4. I manage to get an overview of the game situation from the information received from teammates and the coach	28	3.00	4.00	3.285	0.460	−1.076
		I5. I know at any moment what the other players are doing on the field	28	2.00	4.00	3.428	0.634	−0.438
HB	Ai	I1. I am able to “read” what is happening on the field and “feel” the mood of my teammates	34	2.00	4.00	3.235	0.553	−0.144
		I2. I am able to stop my thoughts while focusing on a certain event, game situation or discussion	34	2.00	4.00	3.352	0.773	−0.921
		I6. When I listen to the coach’s instructions, I am not quickly distracted from my own thoughts or ideas	34	0.00	0.00	2.500	1.051	0.276
	Ae	I3. Even if I focus on what a certain player is doing, I manage to look at things as a whole	34	1.00	4.00	2.647	0.848	−0.667
		I4. I manage to get an overview of the game situation from the information received from teammates and the coach	34	2.00	4.00	3.176	0.796	−1.328
		I5. I know at any moment what the other players are doing on the field	34	0.00	4.00	2.617	1.101	−0.531
VG	Ai	I1. I am able to “read” what is happening on the field and “feel” the mood of my teammates	30	2.00	3.00	2.800	0.406	0.527
		I2. I am able to stop my thoughts while focusing on a certain event, game situation or discussion	30	2.00	4.00	2.800	0.664	−0.634
		I6. When I listen to the coach’s instructions, I am not quickly distracted from my own thoughts or ideas	30	1.00	4.00	2.933	0.784	0.993
	Ae	I3. Even if I focus on what a certain player is doing, I manage to look at things as a whole	30	2.00	4.00	3.133	0.628	−0.321
		I4. I manage to get an overview of the game situation from the information received from teammates and the coach	30	2.00	4.00	3.266	0.691	−0.770
		I5. I know at any moment what the other players are doing on the field	30	2.00	4.00	2.600	0.621	−0.534
VB	Ai	I1. I am able to “read” what is happening on the field and “feel” the mood of my teammates	28	2.00	4.00	3.142	0.524	0.705
		I2. I am able to stop my thoughts while focusing on a certain event, game situation or discussion	28	2.00	4.00	3.357	0.621	−0.554
		I6. When I listen to the coach’s instructions, I am not quickly distracted from my own thoughts or ideas	28	2.00	4.00	3.214	0.686	−0.749
	Ae	I3. Even if I focus on what a certain player is doing, I manage to look at things as a whole	28	2.00	4.00	3.000	0.860	−1.678
		I4. I manage to get an overview of the game situation from the information received from teammates and the coach	28	3.00	4.00	3.357	0.487	−1.732
		I5. I know at any moment what the other players are doing on the field	28	2.00	4.00	3.214	0.568	−0.062
BG	Ai	I1. I am able to “read” what is happening on the field and “feel” the mood of my teammates	29	2.00	4.00	3.000	0.845	−1.615
		I2. I am able to stop my thoughts while focusing on a certain event, game situation or discussion	29	2.00	4.00	3.241	0.786	−1.198
		I6. When I listen to the coach’s instructions, I am not quickly distracted from my own thoughts or ideas	29	0.00	4.00	2.275	0.996	−0.003
	Ae	I3. Even if I focus on what a certain player is doing, I manage to look at things as a whole	29	1.00	4.00	2.482	0.737	−0.089
		I4. I manage to get an overview of the game situation from the information received from teammates and the coach	29	1.00	4.00	2.862	1.059	−0.958
		I5. I know at any moment what the other players are doing on the field	29	1.00	4.00	2.344	0.973	−1.193
BB	Ai	I1. I am able to “read” what is happening on the field and “feel” the mood of my teammates	28	2.00	4.00	3.142	0.650	−0.486
		I2. I am able to stop my thoughts while focusing on a certain event, game situation or discussion	28	2.00	4.00	3.142	0.848	−1.566
		I6. When I listen to the coach’s instructions, I am not quickly distracted from my own thoughts or ideas	28	1.00	4.00	2.857	0.848	−0.034
	Ae	I3. Even if I focus on what a certain player is doing, I manage to look at things as a whole	28	1.00	4.00	3.071	0.899	0.132
		I4. I manage to get an overview of the game situation from the information received from teammates and the coach	28	2.00	4.00	3.071	0.813	−1.463
		I5. I know at any moment what the other players are doing on the field	28	1.00	4.00	3.071	0.978	−0.658

HG—handball group girls; HB—handball group boys; VG—volleyball group girls; VB—volleyball group boys; BG—basketball group girls; BB—basketball group boys; N—numar subiecti; Min.—minimim; Max.—maximum, SD—standard deviation, Ai—Internal attention; Ae—External attention.

**Table 9:** ANOVA (analysis of variance) between groups for the questionnaire for the assessment of attentional style in athletes (QASA).

Group	Attentional Style	∑	df	MS	F	*p*
Girls	Internal attention	10.763	2	5.382	3.619	0.031
	External attention	19.895	2	9.447	3.449	0.000
Boys	Internal attention	12.643	2	6.321	3.264	0.043
	External attention	22.149	2	11.074	3.952	0.023

∑—sum of squares; df—degrees of freedom; MS—mean square; F—test value; *p*—probability level.

**Table 10:** Multiple comparisons LSD—at questionnaire for the assessment of attentional style in athletes (QASA).

Gender	Attentional Style	Sports	Sports	ΔX	SE	*p*	95% CI	
							Lower	Upper
Female	Ai	Handball	Volleyball	−0.533 *	0.320	0.021	−1.170	0.103
			Basketball	−0.862 *	0.323	0.009	−1.504	−0.219
		Volleyball	Basketball	−0.328 *	0.317	0.034	−0.960	0.302
	Ae	Handball	Volleyball	1.214 *	0.412	0.004	0.394	2.034
			Basketball	2.524 *	0.415	0.000	1.697	3.351
		Volleyball	Basketball	1.310 *	0.408	0.002	0.497	2.123
Male	Ai	Handball	Volleyball	−0.890 *	0.355	0.014	−1.597	−0.184
			Basketball	−0.248 *	0.355	0.048	−0.953	0.458
		Volleyball	Baschet	0.642 *	0.372	0.037	−0.096	1.382
	Ae	Handball	Volleyball	−1.159 *	0.427	0.008	−2.008	−0.310
			Basketball	−0.802 *	0.427	0.044	−1.651	0.046
		Volleyball	Basketball	0.357 *	0.447	0.027	−0.532	1.246

* The mean difference is significant at the 0.05 level. ΔX—the difference of the arithmetic means between groups; SE—standard error, *p*—statistical significance values, CI—interval of confidence; Ai—internal attention; Ae—external attention.

**Table 11:** Descriptive statistics of the results of the questionnaire to identify the weights of the qualities of attention according to the characteristics of the practiced sport.

Group	Item	N	Min.	Max.	Mean	SD	Kurtosis
HG	I1—volume	28	2.00	5.00	3.428	0.835	−0.149
	I2—stability	28	2.00	5.00	3.642	0.911	−0.877
	I3—concentration	28	3.00	5.00	4.071	0.813	−1.463
	I4—distributivity	28	3.00	5.00	3.714	0.712	−0.830
	I5—mobility	28	3.00	5.00	4.071	0.899	−1.804
	I6—distraction	28	1.00	4.00	2.535	0.692	0.096
HB	I1—volume	34	3.00	5.00	3.735	0.709	−0.862
	I2—stability	34	2.00	5.00	3.382	0.779	−0.004
	I3—concentration	34	3.00	5.00	3.735	0.709	−0.862
	I4—distributivity	34	3.00	5.00	3.794	0.640	−0.523
	I5—mobility	34	3.00	5.00	4.147	0.821	−1.457
	I6—distraction	34	2.00	5.00	3.205	0.977	−0.482
VG	I1—volume	30	2.00	5.00	3.700	1.055	−1.396
	I2—stability	30	2.00	5.00	3.800	0.924	−1.336
	I3—concentration	30	2.00	5.00	3.366	0.850	−0.334
	I4—distributivity	30	2.00	5.00	3.366	1.033	−1.050
	I5—mobility	30	2.00	5.00	3.533	0.860	−0.629
	I6—distraction	30	1.00	4.00	2.600	0.770	−0.152
VB	I1—volume	28	2.00	5.00	3.857	1.078	−1.632
	I2—stability	28	3.00	5.00	4.071	0.940	−1.933
	I3—concentration	28	2.00	5.00	3.571	0.959	−0.919
	I4—distributivity	28	2.00	5.00	3.571	1.033	−1.140
	I5—mobility	28	3.00	5.00	3.750	0.927	−1.677
	I6—distraction	28	1.00	5.00	2.821	0.862	0.420
BG	I1—volume	29	3.00	5.00	4.000	0.925	−1.905
	I2—stability	29	2.00	5.00	3.586	0.907	−0.833
	I3—concentration	29	2.00	5.00	3.517	0.949	−0.833
	I4—distributivity	29	3.00	5.00	4.034	0.944	−1.956
	I5—mobility	29	2.00	5.00	3.965	0.944	−1.334
	I6—distraction	29	1.00	3.00	2.413	0.732	−0.566
BB	I1—volume	30	2.00	5.00	3.533	1.008	−1.010
	I2—stability	30	2.00	5.00	3.933	1.014	−0.879
	I3—concentration	30	2.00	5.00	3.333	0.844	−0.200
	I4—distributivity	30	2.00	5.00	3.800	1.030	−0.948
	I5—mobility	30	2.00	5.00	4.233	1.040	−0.082
	I6—distraction	30	1.00	5.00	2.866	1.008	0.173

HG—handball group girls; HB—handball group boys; VG—volleyball group girls; VB—volleyball group boys; BG—basketball group girls; BB—basketball group boys; N—numar subiecti; Min.—minimim; Max.—maximum; SD—standard deviation.

**Table 12:** ANOVA (analysis of variance) between groups in the questionnaire to identify the weights of attention qualities according to the characteristics of the practiced sport.

Group	∑	df	MS	F	*p*
Girls	279.121	2	139.560	12.430	0.000
Boys	157.982	2	78.991	6.968	0.002

∑—sum of squares; df—degrees of freedom; MS—mean square; F—test value; *p*—probability level.

**Table 13:** Multiple comparisons LSD –at questionnaire to identify the weights of attention qualities according to the characteristics of the practiced sport.

Gender	Team Sports		ΔX	SE	*p*	95% CI	
						Lower	Upper
Female	Handball	Volleyball	4.361 *	0.880	0.000	2.610	6.113
		Basketball	1.821 *	0.895	0.045	0.040	3.602
	Volleyball	Basketball	−2.540 *	0.880	0.005	−4.291	−0.789
Male	Handball	Volleyball	1.779 *	0.859	0.041	0.072	3.486
		Basketball	3.129 *	0.843	0.000	1.453	4.805
	Volleyball	Basketball	1.350 *	0.884	0.031	−0.407	3.107

* The mean difference is significant at the 0.05 level. ΔX—the difference of the arithmetic means; SE—standard error; *p*—statistical significance values.

## Data Availability

The datasets generated and analyzed during the current study are not publicly available for reasons of privacy.

## Abbreviations

QASA	questionnaire for the assessment of focus of attention in athletes
QAQCS	questionnaire to identify the weights of attention qualities according to the characteristics of the practiced sport
HG	handball group girls
HB	handball group boys
VG	volleyball group girls
VB	volleyball group boys
BG	basketball group girls
BB	basketball group boys
